# Detecting Resistance to Therapeutic ALK Inhibitors in Tumor Tissue and Liquid Biopsy Markers: An Update to a Clinical Routine Practice

**DOI:** 10.3390/cells10010168

**Published:** 2021-01-15

**Authors:** Paul Hofman

**Affiliations:** 1 Laboratory of Clinical and Experimental Pathology, Université Côte d’Azur, CHU Nice, FHU OncoAge, Pasteur Hospital, 30 Avenue de la Voie Romaine, BP69, CEDEX 01, 06001 Nice, France; hofman.p@chu-nice.fr; Tel.: +33-4-92-03-88-55; Fax: +33-4-92-88-50; 2 Hospital-Integrated Biobank BB-0033-00025, Université Côte d’Azur, CHU Nice, FHU OncoAge, 06001 Nice, France

**Keywords:** liquid biopsy, lung cancer, *ALK*, resistance, targeted therapy

## Abstract

The survival of most patients with advanced stage non-small cell lung cancer is prolonged by several months when they are treated with first- and next-generation inhibitors targeting *ALK* rearrangements, but resistance inevitably emerges. Some of the mechanisms of resistance are sensitive to novel ALK inhibitors but after an initial tumor response, more or less long-term resistance sets in. Therefore, to adapt treatment it is necessary to repeat biological sampling over time to look for different mechanisms of resistance. To this aim it is essential to obtain liquid and/or tissue biopsies to detect therapeutic targets, in particular for the analysis of different genomic alterations. This review discusses the mechanisms of resistance to therapeutics targeting genomic alterations in *ALK* as well as the advantages and the limitations of liquid biopsies for their identification.

## 1. Introduction

The therapeutic algorithm for advanced stage non-small cell lung cancer (NSCLC) includes immunotherapy, with or without chemotherapy, chemotherapy or therapy targeting alterations in *EGFR, ALK, ROS1, BRAF, RET, NTRK* or *HER2*. Hence, according to international guidelines, it is mandatory to systematically look for the presence of at least the *EGFR*, *BRAF*, *ALK* and *ROS1* genomic alterations in advanced and metastatic lung adenocarcinoma [1]. Subsequent to the increase in the availability of this therapeutic arsenal, different successive lines of targeted therapy have been envisaged to adapt to the molecular modifications made by cancer cells. In fact, after an initial phase of tumor response, which can be more or less complete, the tumor inevitably induces mechanisms of resistance. However, a number of these mechanisms are sensitive to new therapeutics [1]. Their detection relies essentially on successive genetic analyses using nucleic acids from tumors [1]. These analyses can be done with a tissue biopsy, a cytological sample and/or a liquid biopsy (LB). Therefore, the question of repeating a tissue biopsy or of combining this biopsy with a LB or of follow-up with LB alone can be raised.

*ALK* rearrangements are found in 3–5% of patients with NSCLC [2]. Interestingly, *ALK* rearrangements occur mainly in adenocarcinoma, but though rare can be detected in other histological subtypes of lung cancers such as squamous cell carcinoma and rarely in pulmonary lymphoepithelioma-like carcinoma [2,3,4,5,6,7,8]. The detection of an *ALK* rearrangement of a patient with advanced stage NSCLC is performed with a tissue sample by immunohistochemistry (IHC), fluorescence in situ hybridization (FISH), targeted polymerase chain reaction (PCR) or next-generation sequencing (NGS) [9,10]. However, more recently, detection of molecular targets with a LB, notably using a NGS approach is proposed before providing treatment [11,12]. When an *ALK* rearrangement is identified, targeted treatment with a specific first-, second- or third-generation tyrosine kinase inhibitor (TKI) can be proposed [10,13,14,15]. Since the introduction of the first-generation ALK TKI crizotinib, more selective and better central nervous system penetrant second-generation (alectinib, brigatinib, ceretinib) and third-generation (lorlatinib) can be used in daily practice, and many others such as ensartininb are in development [16,17,18]. Globally, these different ALK TKI can be also classified according to their binding sites [15]. Hence different complex classes of small molecule protein kinase inhibitors, namely types I, II and III, have been defined initially [15]. The type I inhibitor binds with the ATP pocket of the active conformation of the kinase, the type II inhibitor binds to an inactive conformation of the enzyme and the type III inhibitor is a non-ATP competitive antagonist. Finally, the allosteric inhibitors bind to a site different from the active site. This refers to some ligands binding outside of the ATP-binding pocket of some protein kinases [15]. Therefore, several molecules are now available and their efficacy has been compared in clinical trials, in particular the ALEX (crizotinib versus alectinib), ALTA (crizotinib versus brigatinib) or CROWN (crizotinib versus lorlatinib) trials [19,20,21]. Indeed, the therapeutic landscape for advanced *ALK*-positive NSCLC is rapidly evolving [10,13,14]. Second-generation ALK TKIs are widely used in the crizotinib-resistant setting and have become the preferred first-line therapy for patients with advanced disease [9,10]. Recently, the third-generation TKI lorlatinib demonstrated clinical efficiency in previously treated patients, including those who did not respond to one or more of the second-generation TKIs, leading to regulatory approval of lorlatinib in the USA and Japan [22]. It is noteworthy that *ALK* rearrangement can induce PD-L1 upregulation in NSCLC and this finding suggested the potential use and efficacy of anti-PD-L1 molecules in these patients [23]. Hence, different clinical trials evaluated the potential clinical benefits of immune check-point inhibitors (ICIs) in *ALK* positive lung cancer patients [24,25,26,27,28]. Different combinations of ICIs and *ALK* inhibitors showed promises in some series [24,25,28]. However, other studies demonstrated the absence of benefit of these combinations in *ALK* rearranged NSCLC patients [26,27].

The mechanisms of resistance targeting *ALK* rearrangements include genomic alterations, in particular but not exclusively, mutations in *ALK* that differ according to the treatment, as well as other cellular mechanisms such as transformation into a small cell lung carcinoma or epithelial-mesenchymal transition (EMT) [29,30,31,32]. Some of these mechanisms are detected with tissue and/or LB and can lead to administration of a targeted therapeutic [9,11,12,13,14].

This review examines the advantages and limitations of LB for the detection of genomic alterations in *ALK* in advanced stage NSCLC as well as the different mechanisms of therapeutic resistance.

## 2. The Pros and the Cons of Assessment of Circulating Tumor Cells or Circulating Free DNA

It has been recognized for many years that genomic alterations can be detected with different components of blood, most notably circulating tumor cells (CTCs) and circulating free DNA (cf-DNA), and that they can point to the choice of targeted therapy for administration to patients with late stages lung adenocarcinoma [33,34,35]. Discussion has frequently been oriented to better assessing CTCs or cf-DNA in thoracic oncology, but it is currently recognized that circulating free nucleic acid in the plasma is the best substrate for sequencing analyses [33,34,35]. Indeed, some genomic alterations are routinely detected using targeted sequencing or NGS based on the analysis of several hundreds of genes [12,27]. However, CTC assessment is not yet deployed in the daily practice in thoracic oncology field [34]. Indeed, several constraints exist when using the detection of CTCs for genomic alteration assessment in routine clinical practice, (i) the existence and the selection of a robust, sensitive and specific method for reproducible results obtained from different studies, (ii) the difficulty to standardize the pre analytical phase (i.e., the management of the whole blood sample since the veinule puncture, including the type of transport buffer, the time duration for the transfer to the biology laboratory, and the temperature of the sample until the analysis) leading to the detection and characterization of CTCs, (iii) the large variability of the number of CTCs for different lung tumors among different patients, (iv) the heterogenous phenotype and genotype of the different CTCs, (v) the cost, (vi) the difficulty to obtain accreditation according to the ISO 15189 norm, for example, for methods and tests, and, (vii) the turnaround time to obtain results compatible with the care of patients with lung cancer [33,34,35]. However, the genomic analyses from CTC may be complementary to those obtained from circulating free nucleic acids leading to an increased sensitivity of the LB approach, notably for detection of amplifications and gene fusions [34]. Indeed, FISH of CTCs identified on the surface of filters after blood filtration led to the detection of different gene mutations but also gene amplifications and rearrangements, notably in *ALK*, *ROS1*, *MET* and *RET* [35] Conversely, the evaluation of genomic alterations with cf-DNA extracted form blood of late stage lung cancer patients has been routinely performed for many years [33]. Different approaches have been Food and Drug Administration (FDA) and European Medicines Agency (EMA) approved and many European laboratories have accredited these tests in their practice, notably according to the ISO 15189 norm. The methods are automatized and standardized thus providing robust, specific and sensitive results for different genomic alterations. However, the evaluation of certain gene amplifications and/or fusions (notably of *MET*, *RET* and *ALK*) can be more difficult with CTCs and/or tissue biopsies, in particular when the quality and/or quantity of the extracted nucleic acids is low [31,33,34,35].

## 3. Detection of *ALK* Rearrangements at Diagnosis and on Tumor Progression

**Figure 1:**
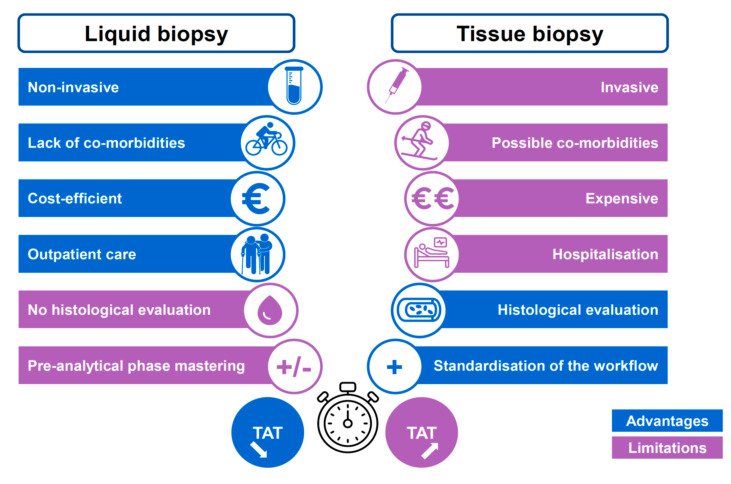
Main advantages and limitations of the LB and of the tissue biopsy in thoracic oncology. TAT, turnaround time.

**Figure 2:**
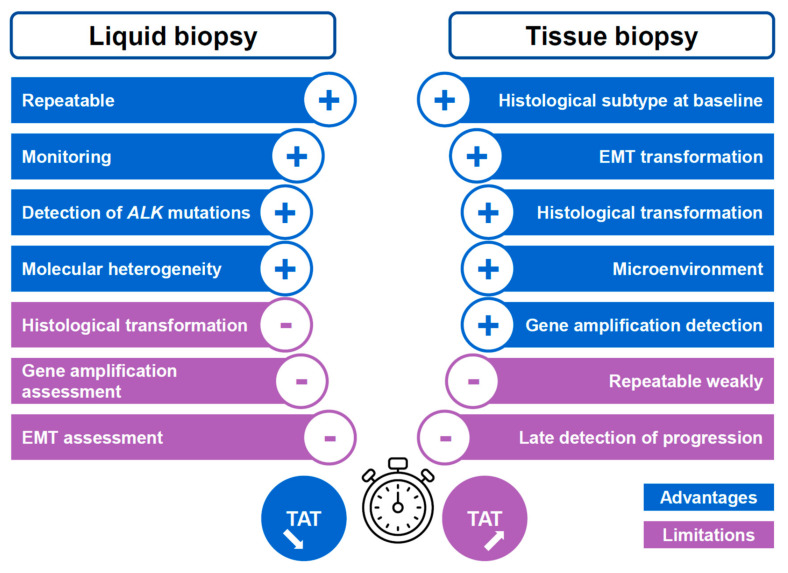
Main advantages and limitations of a liquid biopsy and a tissue biopsy for *ALK*-positive patients. TAT, turnaround time.

According to international guidelines, the initial detection of *ALK* rearrangements is done with IHC, FISH, targeted PCR or NGS using a tissue biopsy or cytological samples showing a non-squamous lung adenocarcinoma [1,9,10,36]. These approaches have variable sensitivities and specificities but overall are satisfactory, where each has its advantages and limits. The advantages and limitations of LB *versus* tissue biopsy practices in oncology are well-known nowadays (Figure 1). More specifically, the assessment of the *ALK* status with LB performed both at baseline or on tumor progression in patients treated with ALKTKI present certain advantages compared to tissue biopsies approaches (Figure 2). The performance of LB for detection of genomic alterations, before administration of treatment, has been evaluated and a recent study reported an almost identical sensitivity to that of a tissue biopsy [11]. In this study, called the Noninvasive versus Invasive Lung Evaluation (NILE) trial, analysis of the cf-DNA detected all guideline biomarkers (*EGFR*, *ALK*, *ROS1*, *BRAF*, *RET*, *MET*, *ERBB2*) included in the International Association for the Study of Lung Cancer (IASLC) guidelines at a rate similar to that of a tissue-based assay [11]. Importantly, while the complete genotype of around 30% of lung cancer patients could not be obtained with a tissue biopsy, because not enough tumor tissue was available for analysis, a LB provided this information. Finally, in this study, the positive predictive value for cf-DNA for tissue genotyping, including *ALK* rearrangements was 100% [11]. A recent study into the analysis of cf-DNA performed on more than 8300 late stage NSCLC patients showed that gene fusions (on *ALK, RET, ROS1*) were identified at baseline in more than 2.3% cases, underlying that LB testing in these patients could be a powerful tool to detect some genomic alterations, and so could be a primary option, at least for patients with an incomplete tissue biopsy analysis [37]. However, we need to keep in mind that the use of a LB at the time of initial diagnosis has to be considered only in patients who need to receive a molecular analysis, notably when: the amount of tissue is low, the percentage of tumor cells in a biopsy is low, long delays in diagnosis are expected due to the organization as well as to the tissue biopsy workflow, and finally, if contraindication to performing endoscopy or a transthoracic biopsy exist. However, as stated by the IASLC with respect to the LB statement work, a positive result for a *ALK* rearrangement obtained with a LB is sufficient to administrate ALK-targeted therapy [12]. To assess the *ALK* status, different studies used different technologies for targeted analysis of a gene or large panels of genes [11,38,39,40]. The recent BFAST (Blood First Assay Screening Trial) study used NGS panels that showed the excellent sensitivity and specificity of LB for the detection of *ALK* rearrangements as compared to the results obtained with tissue samples [11,41]. The *ALK*-positive tumors were treated based on the results obtained with LB markers only and showed a positive response to ALK inhibitors [41]. This study merits a number of comments, (i) changes in the *ALK* status were detected with a centralized NGS analysis with a panel of several genes, and, (ii) the number of patients included was relatively small [41]. Therefore, an additional independent study may be required to validate the results. Moreover, different studies revealed a lower level of detection of *ALK* rearrangements in LB compared to detection in matched tissue biopsies of patients [39,42,43]. The persistence or reappearance in tumors of an *ALK* rearrangement when on targeted treatment is sometimes associated to initial resistance or tumor progression and may be investigated using a new tissue biopsy or even a LB. It is noteworthy that clinical courses of *ALK* positive tumors treated with ALK inhibitors vary for a patient to another one. In this context, *ALK* amplifications or resistance mutations or activation of bypass signaling pathways emerge now in a poorly predictive manner. In this context the risk detected at baseline can be affected by specific molecular parameters such as the presence of *ALK* fusion variant, and, importantly to the presence of *TP53* co-mutations [44,45,46,47].

The detection of an *ALK* rearrangement using blood is performed with circulating free nucleic acids in the plasma using targeted approaches or NGS [11,39,42]. Other approaches have been developed including detection and characterization of CTCs isolated by filtration of blood and the identification of the *ALK* status by IHC and/or FISH [48,49,50]. However, multi-centric comparative and validation studies are necessary to define the sensitivity and specificity of these approaches. Compared to approaches using ccf-DNA, the difficulties associated with mastering the pre-analytical (e.g., the different steps from blood taken samples until the nucleic acids extraction) and analytical phases after filtration of blood may make difficult the development of CTC analyses in routine practice [51,52]. Finally, a few studies recently highlighted the interest of evaluating the *ALK* status with platelets [53,54]. In fact, potential *ALK* rearranged tumor cells release RNA into the blood stream by a variety of different vehicles, such as exosomes. Then, these different circulating vehicles are able to transfer tumor-derived RNA into platelets [53]. Hence, *ALK* status can be assess using nucleic acids extracted from these platelets [53]. However, it is interesting to see that this approach, which seems quite easy to set up, is not currently used in the daily practice.

## 4. Mechanisms of Resistance to Treatment with Tyrosine Kinase Inhibitors Targeting *ALK*

Mechanisms of resistance inevitably emerge after a variable period of time of treatment with TKIs targeting *ALK* [55]. Almost all the patients treated with crizotinib developed resistance within 12 to 24 months and showed disease progression. Importantly, approximatively 50% of the patients developed a central nervous system disease during the course of treatment [56]. Second-generation ALK TKIs, including ceritinib, alectinib, and brigatinib, overcome the acquired resistance of crizotinib-pretreated ALK-positive tumors. Notably, brigatinib has been reported to overcome the crizotinib-resistant *ALK* G1202R mutation in a preclinical model and was active in vitro against many other ALK kinase domain mutations such as V1180L, L1196M, L1152R/P, C1156Y, E1210K, G1269A, and I1171S/T. Finally, the third-generation ALK TKIs lorlatinib, entrectinib, and ensartinib showed very promising results in terms of clinical activity and safety, and harbor a high target potency with the widest spectrum of activity toward crizotinib resistance mutations [10,13,14]

Mechanisms of resistance may concern, (i) *ALK* mutations, or, (ii) mechanisms independent of *ALK*, touching or not other genomic alterations [57,58,59,60,61]. Depending on the TKI, and, the sequence targeted by the treatment, some mechanisms of resistance appear more frequently. Initial mechanisms of resistance to therapeutic inhibitors targeting *ALK* are uncommon. These mechanisms may concern, at least in theory, the same cellular mechanisms that emerge at recurrence or progression of *ALK*-positive tumors of patients treated with TKI to ALK, in particular to mutations in *ALK* or even certain fusions of *ALK* or even more to false resistance linked to false positive results on interpretation of *ALK* FISH [62,63,64].

### 4.1. Mutations in ALK

These mutations can be identified by different techniques when using plasma, which mainly, but not exclusively, involve NGS approaches using more or less enlarged panels [30,39,65]. Depending on the TKI administered and the therapeutic sequence that indicates which TKI is to be prescribed, the resistance mutations in *ALK* can differ and/or emerge more frequently [30,60,66,67,68]. These mutations appear in about 20–30% of patients treated with crizotinib and in more than 50% of patients treated with a second-generation ALK TKI [24]. The first identification of resistance mutations in *ALK* (L1196M, G1269A, C1156Y, L1152R, etc.) was described after treatment of patients with crizotinib. The mutation L1196M is probably the most well-known resistance mechanism. The *ALK* G1202R mutation has been described in a number of studies to confer a high level of resistance of tumors of patients treated with first- and second-generation ALK TKI [30,69,70]. This mutation is rarely found in patients on crizotinib but is frequent after treatment with alectinib or brigatinib [30]. Hence, the G1202R substitution is found in only 2% of crizotinib-resistant patients and in 21–43% of patients after treatment with a second-generation ALK TKI. In a recent study, Noé et al. reported that 48/187 (25.7%) of patients developed a mutation in *ALK* after treatment with crizotinib [61]. The mutations I1171 T/N/S, G1202R, and V1180L in *ALK* appear frequently after treatment with alectinib [71,72]. Fortunately, after progression on a second-generation ALK TKI data from a number of studies reported that third-generation ALK inhibitors showed promise. It is interesting that these mutations are sensitive to novel TKIs to ALK such as lorlatinib and/or ceritinib [72]. Indeed, it seems important to look precisely for these mutations since the sensitivity to ALK TKIs can vary and, depending on the different molecules administered, is often complex to grasp [73]. In a study by Dagogo-Jack et al. a mutation in *ALK* was detected in 46/70 (66%) patients treated with a second-generation TKI [74]. When using tissue and LB for sequencing, a similar proportion of mutation in the same patients (60 and 70%, respectively) was found after treatment with alectinib, but analyses with LB identified more often multiple mutations in *ALK* [74]. A mutation in *ALK* was found in more than 75% of patients treated with lorlatinib who relapsed and more than two mutations were detected in 50% of cases. Among the double mutations, the association of G1202R/L1196M and D1203N/1171N was the most frequent [74]. These mutations were most often detected with nucleic acids isolated from plasma but recent studies showed that identification was also feasible with isolated CTCs [75]. Resistance mechanisms implicating amplification of *ALK* are less frequent than mutations in *ALK* and may or may not be associated with the latter [69,76].

### 4.2. MET Genomic Alterations

Amplification in *MET* is a rare genomic alteration detected in NSCLC. In a large cohort of 2694 NSCLC screened by NGS, a primary or acquired *MET* amplification were detected in 3.27 and 16.04% of tumor tissue collected from lung cancer patients, respectively [77]. Interestingly patients having a tumor with a copy number greater than 4 seemed to have a longer progression free survival (PFS) after crizotinib treatment. Moreover, no significant differences in PFS were observed between patients with primary or acquired *MET* amplifications. Amplification in *MET*, and more rarely rearrangement in *MET*, emerge during treatment with ALK TKI [78,79]. So, about 15% of patients treated with new generation inhibitors develop *MET* amplifications [78]. Moreover, in the study by Dagogo-Jack et al., around 12 and 22% of tissue biopsies from patients progressing on second-generation inhibitors or lorlatinib, respectively showed a *MET* amplification [78]. In the same study lung cancer patients treated with a second-generation ALK inhibitor in a first-line setting were more likely to develop a *MET* amplification than those who had received next-generation ALK inhibitors after crizotinib. Moreover, patients with detected *MET* amplifications in the tumor tissue can also be treated with crizotinib [79]. The detection of *MET* amplification is easier in DNA extracted from tissue than from plasma, this latter showing globally a lower sensitivity, even if some study demonstrated that the detection of copy number variation (CNV) could be similar in blood and in tissue [80].

### 4.3. Other Genomic Alterations

These genomic anomalies emerge at a frequency that depends on the TKI. Several genes and mechanisms of resistance are concerned including activation of *EGFR* or over expression of NRG1 (in the case of treatment with crizotinib) [81,82]. Other genomic anomalies concern *PIK3CA*, amplifications in *KIT*, activation of IGF-1R or of SRC, mutations or amplifications in *DDR2, BRAF, NRAS* or *FGFR2* for example [30,69,83,84]. Recently, Makimoto et al. found a lot of mutations in *ALK*-rearranged tumors associated with early resistance to alectinib [85]. *ALK* fusion gene copy number abnormalities include copy number gain (CNG) and gene deletion. The resistance mechanism involving an abnormal *ALK* gene copy number is similar to that of a gene mutation, increasing the activity of the kinase. The CNG means that the average number of the rearranged genes in lung cells are more than tripled. The analysis of the genes in crizotinib-resistant patients found that the *ALK* fusion gene copy number increased dramatically, confirming that CNG is to blame for the resistance at least in in vitro experiments [86]. Most of these genomic alterations can be detectable in LB, even some of them such as *ALK* CNV, can be more difficult in nucleic acids extracted from plasma samples.

### 4.4. Epithelial-Mesenchymal Transition and Histological Transformation

EMT is a phenomenon characterized by a more or less complete loss in expression of epithelial markers by cancer cells including cadherins such as E-cadherin and by strong expression of mesenchymal markers, in particular vimentin [87,88]. Some studies report strong expression of vimentin in tumors resistant to ALK TKI such as ceritinib. The exact molecular mechanisms of this phenomenon and the resistance are still unknown. However, to better understand the mechanisms induced by the treatment, an interesting study looked at the expression of genes involved in EMT associated with crizotinib resistance in treated ALK-positive tumors [89]. In this study, Wei et al. used whole genome sequencing approaches and were able to identify 175 variants in 156 genes that were enriched in crizotinib-resistant tumor samples as compared to matched pre-crizotinib samples [89]. In some patients, pathway analysis revealed an enrichment in nine genes in four EMT pathways. These four pathways concerned proteoglycans in cancer (*ANK2, FASLG, HSPG2, PTPN11, STAT3*, and *VEGFA*), HIF-1 signaling (*ARNT, STAT3*, and *VEGFA*), FoxO signaling (*FASLG, SMAD4*, and *STAT3*), and ECM-receptor interactions (*HSPG2* and *LAMA2*) [89]. Histological transformation of the tumor with the appearance of a small cell carcinoma can occur in a low percentage of patients treated with TKI targeting *ALK* and may be associated with an *ALK* mutation and/or a loss in RB [90,91,92]. Though uncommon, transformation of the tumor with the appearance of a squamous cell carcinoma has also been described [93]. Taken together, and concerning these mechanisms of resistance, it is well admitted that LB cannot be currently of strong help.

## 5. Advantages and Limitations of Liquid Biopsy in *ALK* Positive Lung Cancer Patients

The LB and the tissue biopsy have advantages, but also limitations for each of them in patients demonstrating an *ALK*-positive lung tumor (Figure 2). In this regard, they can be complementary approaches.

### 5.1. Advantages of a Liquid Biopsy

As mentioned above, the BFAST study and the NILE trial reported excellent agreement for the detection of certain genomic alterations, including *ALK* rearrangements [11,41]. Compared to a tissue biopsy, blood sampling is noninvasive, painless, can be repeated over time, or when the biological sample is not of good enough quality for analysis due to a defective pre-analytical phase. Thus, monitoring patients on targeted anti-ALK therapy is much more feasible with repeated blood sampling than with tissue sampling, in particular for fragile and/or aged patients [32,58,94,95,96]. A renewed tissue sample obtained during broncho-endoscopy or trans-thoracic core biopsy or biopsy at a metastatic site can lead to complications or, though rare, death [97]. Certain metastatic bone biopsies require decalcification, which can degrade the nucleic acids before the molecular analysis and may lead to a false-negative result. Therefore, at the time of a bone biopsy a LB and a tissue biopsy should also be considered. Moreover, a LB does not require hospitalization or medical expertise as does a tissue biopsy obtained during endoscopy and radiological imagery. Moreover, a LB examines the molecular heterogeneity of a cancer that is present in different metastatic sites and favors overall evaluation of the molecular status of this cancer. Thus, several mechanisms of resistance to ALK TKI can exist for an individual patient and can be evaluated at different metastatic sites using LB, which are not accessible with repeated tissue biopsies [55,78]. Finally, one of the advantages of a LB concerns the early detection of recurrence or of residual disease, while radiological examination at the same moment in time may not detect an accessible tissue target for molecular analysis [98]. It is of interest to highlight that for non-adenocarcinoma lung tumors, notably for squamous cell carcinoma, but also for other rare histological subtypes such as pulmonary lymphoepithelioma-like carcinoma, the detection of an *ALK* rearrangement is not mandatory with tissue biopsies [1]. However, though uncommon these tumors can present an ***ALK*** rearrangement [3,4,5,6,7,8]. Moreover, these rare tumors showed response to first- and second-generation ALK-TKI [6,99,100]. Hence, is it possible to screen systematically for *ALK* fusions with tissue biopsies not only from lung adenocarcinomas, but also from other lung cancer subtypes? [5]. In this context, the use of LB for detection would be probably easier in routine practice. Finally, some discrepancies between the assessment of the *ALK* status using ALK IHC and ALK FISH can exist with tissue biopsies [101,102,103]. Notably some borderline *ALK* FISH results have been described and make treatment decisions concerning ALK TKI administration a challenge in these latter cases [101]. It may be of interest to use a LB for these patients to look for and confirm the presence of an *ALK* rearrangement.

### 5.2. Limitations of Liquid Biopsies

Over the years the limitations of LB have diminished due to technological developments such as improvement in the methods of extraction of nucleic acids from plasma, increases in the sensitivity of the analytical systems, which thereby require smaller and smaller amounts of biological material, and the introduction of new panels of genes. However, several points must be raised in highlighting the need to still consider the tissue biopsy as the gold standard in the majority of situations. The sensitivity of a LB can be lower than that of a tissue biopsy for biological reasons. Some small-sized or slow-growing tumors or even some metastatic sites such as the central nervous system, release only small amounts of nucleic acid into the blood. It is of interest to note that the sensitivity of assessment of the *ALK* status is much lower with a LB from the blood than with cerebrospinal fluid samples for patients with metastases to the brain [104,105]. According to a study by Auliac et al. around 21% of LB did not provide information to get the *ALK* status [106]. In fact, NGS approaches detected different genomic alterations in cancer cells including single nucleotide variations, insertions and deletions, focal amplifications, gene fusions, copy number alterations and numerical and segmental chromosomal alterations. Studies comparing analyses with NGS obtained with blood or tissue from the same patient are sometimes conflicting. Many studies demonstrated a lower sensitivity of NGS approaches from LB samples for the detection of an *ALK* rearrangement, compared to matched patient tissue biopsies [42]. Moreover, some genomic anomalies including fusions, copy number alterations and some focal amplifications (in particular in *MET* or *ALK*) seem to be more difficult to detect in blood than in tissues, as shown in a number of comparative studies [107,108,109,110]. It is also possible that the lower sensitivity of LB is linked to poorly controlled pre-analytical steps, (i) an inadequate amount of plasma to obtain an optimal amount of nucleic acid for analysis, and, (ii) a too long delay between sampling of blood and centrifugation, resulting in liberation of a lot of nucleic acid of germinal origin (from circulating hematological cells), which limits the analysis of plasma nucleic acids released from tumor cells [111]. In this situation, certain mutations associated with clonal hematopoiesis must be identified, in particular in the elderly, and distinguished from somatic mutations related to the phenomenon of resistance on treatment [112].

Some genomic alterations are more difficult to detect with a LB than with a tissue biopsy, in particular in the case of the poor quality and/or an insufficient amount of nucleic acid, which concerns primarily fusions and amplifications. Thus, fusions in *ROS1, RET, NTRK1* and amplifications in *MET*, *ALK, EGFR,* for example, may not be detected with circulating DNA and RNA, notably during tumor progression under treatment with TKI targeting *ALK* rearrangements. In this context, some authors discussed the possibility to look for *ALK* amplifications or for *ALK* rearrangements using analyses with CTCs, notably FISH approaches [48,49,52]. However, as discussed above, this method can be time consuming and not fast enough for treatment decision making, and, the number of CTCs is variable, sometimes low, depending for example on the tumor burden. Consequently, the possibility of performing a new tissue biopsy on cancer progression, which depends of the primary tumor and/or the metastatic sites, as well as the patient’s general condition, may be open to discussion for the detection of genomic anomalies with NGS and/or FISH. Moreover, only a tissue biopsy can identify the histological signs of transformation into a small cell carcinoma or squamous cell carcinoma or can better and easily determined the presence of EMT. Hence, some studies showed that EMT can be also detected in CTCs having a low or a non-expression of cytokeratins (notably the E-cadherin) and a high expression of different biomarkers such as vimentin [34].

Proficient implementation of the pre-analytical phases of LB is essential to obtain good quality cf-DNA and robust analyses that provide reliable results [113,114]. A number of steps need to be controlled including the volume of blood collected (until 10 to 20 mL of whole blood, according to the size of the genes panels used for NGS), the nature of the tubes used to collect the blood (EDTA when the turnaround time before centrifugation is less than 4 h, or other tubes such as Streck BCT tubes if this turnaround time is longer), the temperature of transport (between 15–25 °C), the delay before centrifugation, the centrifugation speed and protocol used, and storage conditions of the blood or plasma (at least at −20 °C and ideally at −80 °C before nucleic acids extraction) [113,114]. This limits the use of LB compared to tissue biopsies for which the pre-analytical management is now more straight forward and standardized since samples are immediately fixed in formalin at room temperature. Thus, irrespective of the performance of the analytical aspect, the quantity and quality of the nucleic acids that can be obtained from a sample of plasma depend strongly on the pre-analytical phase and the optimization of the different parameters, as well as the tumor burden and the metastatic sites [115]. However, it is noteworthy that sometimes nucleic acids extracted from tissue sample can be of low quantity or of low quality and degraded due for example to the deamination effect induced by the formalin fixative [116,117]

## 6. Conclusions

Currently, when a tumor progresses, clinicians ask themselves if it is necessary or not to look for mechanisms of resistance to TKI that target ALK. Hence the therapeutic strategy indicates almost systematic administration of a second-generation inhibitor and then on further progression the latest generation of inhibitors. This strategy is developed whatever the detection of an ALK mutation in the tumor, since *ALK*-positive lung cancers are sensitive to these inhibitors, even in the absence of an ALK mutation. In addition, according to the recent recommendations of the European Society for Medical Oncology (ESMO), the detection with a new biopsy (liquid or tissue) of mechanisms of resistance on progression is not mandatory and even more, detection does not figure in the recommendations of the National Comprehensive Cancer Network (NCCN) [118,119].

It is pivotal to be aware that tumors progress while on ALK TKI and currently the fact that no mechanism of resistance has been identified for that occurrence constitutes an unmet medical need. We strongly believe that it is essential to look for mechanisms of resistance to treat patients with TKI that target ALK to better understand the pathophysiology and the way ALK-rearranged NSCLC adapt to different therapeutics [120,121]. If a disease progresses during treatment with first-generation and, more often now with second-generation ALK TKI, a LB should be the first choice in evaluating resistance mechanisms. Plasma cf-DNA has a very high specificity for detection of ALK mutations, even if a tissue re biopsy remains another solution. Even if there is still ongoing several gaps for detecting the different resistance mechanisms of ALK inhibitors in ALK positive treated lung cancer patients (notably the histological type transformation of the tumors, some gene amplifications or fusions, etc.), the LB presents currently strong advantages at tumor progression, such as those described above. Moreover, recently it has been demonstrated that combined copy number and targeted mutation profiling can be of strong interest for improving monitoring ALK positive NSCLC, notably for tumors without any detectable mutations [122]. In addition a tissue biopsy should be pursued in cases of suspected small cell lung cancer transformation and also when a LB does not reveal a likely resistance mechanism. Moreover, the knowledge obtained from different analyses that integrate over time tissue and LB will certainly lead to the development of novel therapeutic molecules [123,124]. It is certainly too premature to assert that liquid biopsies will replace tissue biopsies for the evaluation of the status of ALK at the time of diagnosis and/or on progression of all the mechanisms of resistance of a tumor of a patient treated with TKI that target an ALK rearrangement. Thus, to optimize the care of patients with ALK-rearranged tumors expert diagnostics must combine different approaches using several biological sources [125,126,127,128,129]. In this context the integration at the same time of different information obtained from different sources and components of a LB, not only CTCs and cf-DNA, but also circulating plasmatic microRNA and exosomes may in the upcoming years represent a new challenge to better understand the mechanisms of resistance to the different generations of ALK TKI [130,131].
